# Crystal structure of potato 14-3-3 protein St14f revealed the importance of helix I in StFDL1 recognition

**DOI:** 10.1038/s41598-022-15505-y

**Published:** 2022-07-08

**Authors:** Ken-ichi Harada, Kyoko Furuita, Eiki Yamashita, Ken-ichiro Taoka, Hiroyuki Tsuji, Toshimichi Fujiwara, Atsushi Nakagawa, Chojiro Kojima

**Affiliations:** 1 Institute for Protein Research, Osaka University, Suita, Osaka Japan; 2 Kihara Institute for Biological Research, Yokohama City University, Yokohama, Japan; 3 Graduate School of Engineering Science, Yokohama National University, Yokohama, Japan

**Keywords:** NMR spectroscopy, X-ray crystallography, Flowering, Solution-state NMR, X-ray crystallography

## Abstract

In potato (*Solanum tuberosum* L.), 14-3-3 protein forms a protein complex with the FLOWERING LOCUS T (FT)-like protein StSP6A and the FD-like protein StFDL1 to activate potato tuber formation. Eleven 14-3-3 isoforms were reported in potato, designated as St14a-k. In this study, the crystal structure of the free form of St14f was determined at 2.5 Å resolution. Three chains were included in the asymmetric unit of the St14f free form crystal, and the structural deviation among the three chain structures was found on the C-terminal helix H and I. The St14f free form structure in solution was also investigated by nuclear magnetic resonance (NMR) residual dipolar coupling analysis, and the chain B in the crystal structure was consistent with NMR data. Compared to other crystal structures, St14f helix I exhibited a different conformation with larger B-factor values. Larger B-factor values on helix I were also found in the 14-3-3 free form structure with higher solvent contents. The mutation in St14f Helix I stabilized the complex with StFDL1. These data clearly showed that the flexibility of helix I of 14-3-3 protein plays an important role in the recognition of target protein.

## Introduction

The 14-3-3 proteins are ubiquitously expressed in all eukaryotic cells and are involved in the regulation of diverse cellular functions, such as metabolism, signal transduction, cell cycle control, apoptosis, transcription, and protein trafficking^1–3^. The number of 14-3-3 isoforms of animals is 7 in most cases, but the number of 14-3-3 isoforms of plants depends on the species, for example, 15 in *Arabidopsis*^4^, 12 in tomato^5,6^, 17 in tobacco^7^, 11 in potato^8^, and 8 in rice^9,10^. The 14-3-3 proteins, including all isoforms, belong to a single family with structural similarity and are thought to function as homo- or heterodimers^11,12^.

Plant 14-3-3 proteins are divided into two groups based on their gene structures, namely, epsilon and nonepsilon, and the latter is plant-specific^13–15^. Large-scale protein interactomics mapping and mass spectrometry-based studies have revealed more than 300 proteins putatively binding 14-3-3 in plants^16^. In rice, the nonepsilon 14-3-3 protein GF14 is an interactor of Hd3a and OsFD1 proteins. Hd3a is a rice homolog of *Arabidopsis* FLOWERING LOCUS T (FT) protein, which induces flowering^17^. OsFD1 is the basic leucine zipper (bZIP) transcription factor and a typical 14-3-3 binding protein; it possesses a 14-3-3 binding motif with a phosphorylated Ser/Thr residue and it interacts with 14-3-3 at the binding pocket of the phosphate group of phosphorylated proteins^17,18^. In potato, the nonepsilon 14-3-3 protein St14 interacts with the potato homolog of FT protein, StSP6A, and bZIP transcription factor StFDL1 and regulates potato tuber formation^8^.

Crystal structures of 14-3-3 proteins of both animals and plants have been determined, revealing that 14-3-3 proteins form homodimers, with a characteristic cup-like or horseshoe structure possessing positively charged grooves for pSer or pThr^18^. The 14-3-3 proteins have been recognized as possessing a similar and rigid conformation in both free- and complex- forms because the structures of the free- and complex-forms of both human 14-3-3 zeta and sigma were similar^19^. However, recently, the structural difference between free- and complex-forms of human 14-3-3 beta was reported as the open- and closed-structures, respectively^20^. Furthermore, molecular dynamics (MD) simulation of human 14-3-3 sigma showed structural flexibility and suggested the possibility of facilitating the binding of its ligand^21^.

In this work, the free form structure of St14f was determined by X-ray crystallography at 2.5 Å resolution and compared with the other 14-3-3s. The structure of the I helix of St14f was different from others, and the importance of the I helix of St14f on StFDL1 recognition was studied by nuclear magnetic resonance (NMR) and thermal shift assays.

## Results and discussion

### Crystal structure determination of St14f

**Figure 1:**
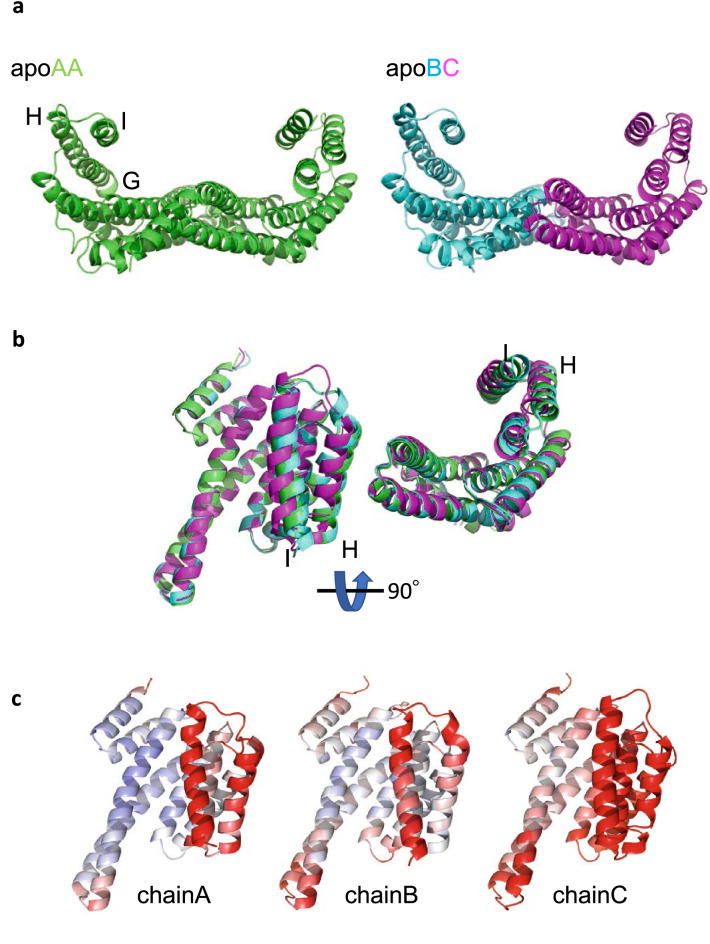
St14f has flexible C-terminal helices H and I. (**a**) Crystal structures of St14f dimers. Three chains (A, B, and C) are involved in the asymmetric unit. Chains A, B, and C are shown as green, cyan, and magenta, respectively. In the crystal, chains AA and BC exist as dimers. The structures of St14f are the same as those of other 14-3-3 proteins, whose monomer consists of nine antiparallel α-helices consisting of a binding site to interact with phospho-serine or threonine motifs. (**b**) Superposed structures of three monomers (chains A, B, and C) of the free St14f form. The N-terminal helices A-G are very similar conformations, and the C-terminal helices H and I show specific conformational differences. (**c**) B-factor of St14f monomers (chains A, B, and C). B-factor coloring is shown as blue-white-red (minimum: 10, maximum: 100). In C-terminal helices H and I, each of the chains showed a high B-factor; in particular, C-terminal helix I showed the largest conformational differences between the three chains. Figures are prepared by PyMol 2.6 (http://www.pymol.org/pymol)^42^.

The crystal structure of the St14f free form was determined at 2.5 Å. Three chains (A, B, and C) are contained in the asymmetric unit, i.e., two conformations of dimers (AA and BC) exist in the crystal (Figs. 1a and S1, Table S1). The St14f dimer has a common characteristic cup-like shape with a disordered C-terminal region of approximately 20 amino acids, similar to other 14-3-3 proteins. The monomer consists of nine antiparallel α-helices possessing a binding site of phosphoserine/threonine. Superposing three chains (A, B, and C) of St14f in the asymmetric unit, we found that the conformation of N-terminal helices A-G was very similar. In contrast, the conformations of the C-terminal helices H and I showed significant differences (Figs. 1b and S1). In addition, helices H and I of all three chains show high B-factors (Figs. 1c and S1), particularly helix I, suggesting conformational flexibility.

### Comparison with other 14-3-3 proteins

**Figure 2:**
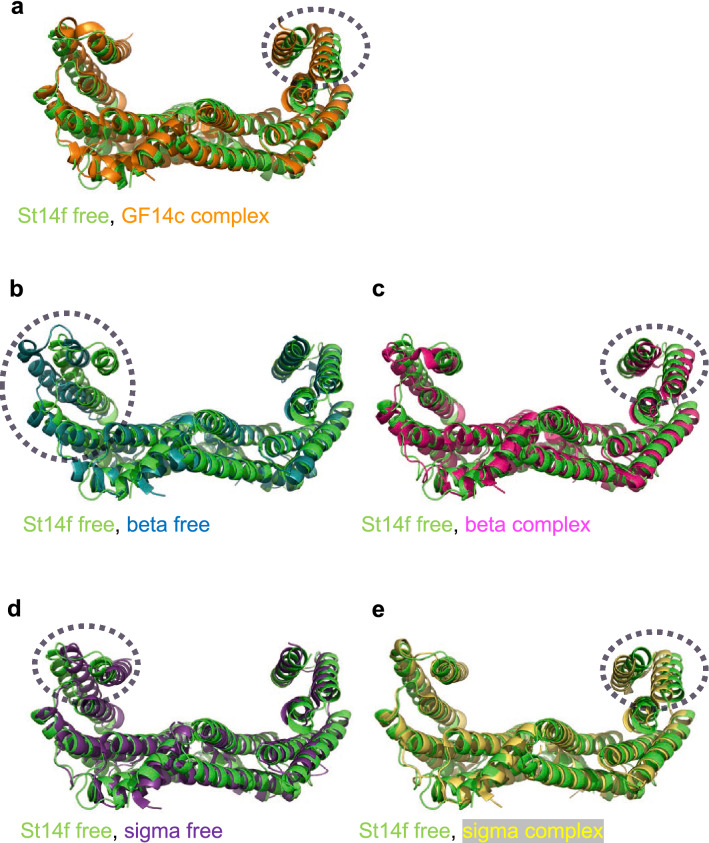
Superposition between the St14f free form (chain AA, green) and other 14-3-3 protein dimers. (**a**) Rice GF14c complex form (orange) reported in PDB ID 3axy, (**b**) human 14-3-3 beta free form (blue) reported in PDB ID 2bq0, (**c**) human 14-3-3 beta complex form (magenta) reported in PDB ID 2c23, (**d**) human 14-3-3 sigma free form (purple) reported in PDB ID 1yz5, (**e**) human 14-3-3 sigma complex form (yellow) reported in PDB ID 1ywt. Figures are prepared by PyMol 2.6 (http://www.pymol.org/pymol)^42^.

To characterize the structure of the St14f free form in detail, it was superposed with other 14-3-3 proteins (free and phosphopeptide complex forms). Superposition was performed using the CCP4 Superpose program, which was the structural alignment of the C-alpha backbone based on secondary structure matching^22^. St14f showed 82% amino acid sequence identity with GF14c, the structure of which was determined in complex with OsFD1 and Hd3a. The whole structure of the GF14c complex form was similar to the structure of the St14f free form, but the local structure in helices H and I was different, and the grooves were narrower than the grooves in the St14f free form (Figs. 2a and S1). The free form of human 14-3-3 beta showed an open structure, and its grooves were wider than the grooves of the St14f free form (Figs. 2b and S1). The grooves of the complex form of the human 14-3-3 beta were narrower than the grooves of the free form of human 14-3-3 beta and wider than the grooves of the St14f free form, especially the local structure in helices H and I, which were different (Figs. 2c and S1). In contrast, the grooves of both free and complex forms of the human sigma were narrower than the grooves of the St14f free form, especially the local structure in helices H and I, which was different (Figs. 2d,e and S1). Helices A-G were superimposed very well in all structures, but helices H and I exhibited conformational differences in many cases (Figs. 2 and S2).

### Solution structure of St14f evaluated by NMR analysis of RDC values

**Figure 3:**
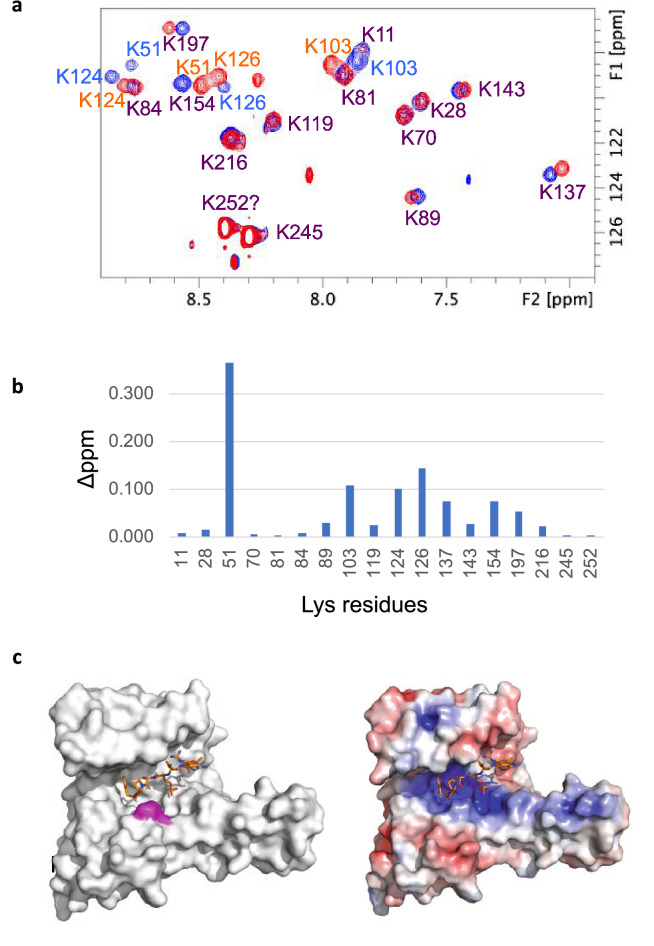
The free and complex forms of St14f in solution could be observed by NMR. (**a**) Overlaid ^15^N-^1^H HSQC spectra of ^15^N Lys-labeled St14f free form (red) and complex form with StFDL1 peptide (blue). The protein concentration was 250 µM in buffer containing 10 mM Tris, pH 7.5, 1 mM DTT, and 10% D_2_O at 298 K. (**b**) Chemical shift changes of St14f in the presence of 0 mM and 1 mM of StFDL1 peptide are represented by a bar graph. (**c**) Mapping of the affected residues on the St14f surface (left) and electrostatic surface potential calculated using APBS, where red and blue correspond to negative and positive electrostatic potentials, respectively (right). The OsFD1 peptide derived from PDBID 3axy is represented by sticks as the model of the StFDL1 peptide. K51 residues with chemical shift changes of > 0.3 are colored magenta. Figures are prepared by TopSpin 3.6 (https://www.bruker.com/en/products-and-solutions/mr/nmr-software/topspin.html) and PyMol 2.6 (http://www.pymol.org/pymol)^42^.

HSQC spectra of ^15^N Lys-labeled St14f were measured with and without StFDL1 peptide (LNRTSpTAPF). Assignment of Lys residues was carried out using point mutation for both free and St14f-StFDL1 complex forms (Supplementary Fig. S3). Chemical shift differences between the free and complex forms were observed for several peaks (Fig. 3a,b). K51 showed the largest chemical shift difference > 0.3 ppm, locating at the positively charged groove where was binding pocket of the phosphate group of the phosphorylated peptide (Figs. 3b,c and S1). K103, K124, and K126 showed the largest chemical shift difference (> 0.1 ppm), in which these residues were located around the peptide-binding site (Supplementary Figs. S1 and S4).

In the crystal, three chains (A, B, and C of St14f) were found and possessed different conformations, named the A, B, and C structures (free form), respectively. To evaluate which conformation of St14f is close to the solution structure, NMR was used. Residual Dipolar Couplings (RDCs) values measured by NMR provide long-distance structural information for proteins in solution. RDC values were experimentally obtained from TROSY and HSQC spectra using Pf1 phage as an aligned medium.

The RDC values of the free and complex St14f were used to assess the crystal structures by PALES software^23^ (Table 1). Two of 18 Lys residues located at the C-terminus were disordered in the crystal structure. Lys residues located at secondary structures were applied to PALES calculation. The residues possessing large B-factors at C alpha atom position > 100 were excluded. The chain C structure (free form) of St14f is not used because the number of Lys residues used for the analysis is limited. The chain B structure of St14f shows the best R and Q values, suggesting that the chain B structure (free form) of St14f is close to the solution structure. Judging from the Q values, the complex structure of the St14f-StFDL1 peptide in solution is different from the free structure of St14f in the crystal, such as the chain A and B structures (free form) of St14f.

**Table 1:** The RDC statistics of St14f are computed by PALES.

	Chain A structure vs NMR RDC data of free	Chain A structure vs NMR RDC data of complex	Chain B structure vs NMR RDC data of free	Chain B structure vs NMR RDC data of complex
DATA N^a^	13	13	13	13
RMS	2.226	0.937	1.685	1.123
Chi^2^	64.436	11.403	36.909	16.383
CORR R^b^	0.877	0.913	0.933	0.872
Q SAUPE^c^	0.174	0.189	0.115	0.261

RDCs were obtained by HSQC-TROSY pairs. Three chains A and B in the St14f crystal were used for the PALES analysis.

^a^The number of residues which gave experimental RDC values.

^b^Pearson’s linear correlation coefficient between experimental and best-fitted RDC values.

^c^Cornilescu's quality factor between experimental and best-fitted RDC values.

### The flexibility of Helix I of St14f

**Figure 4:**
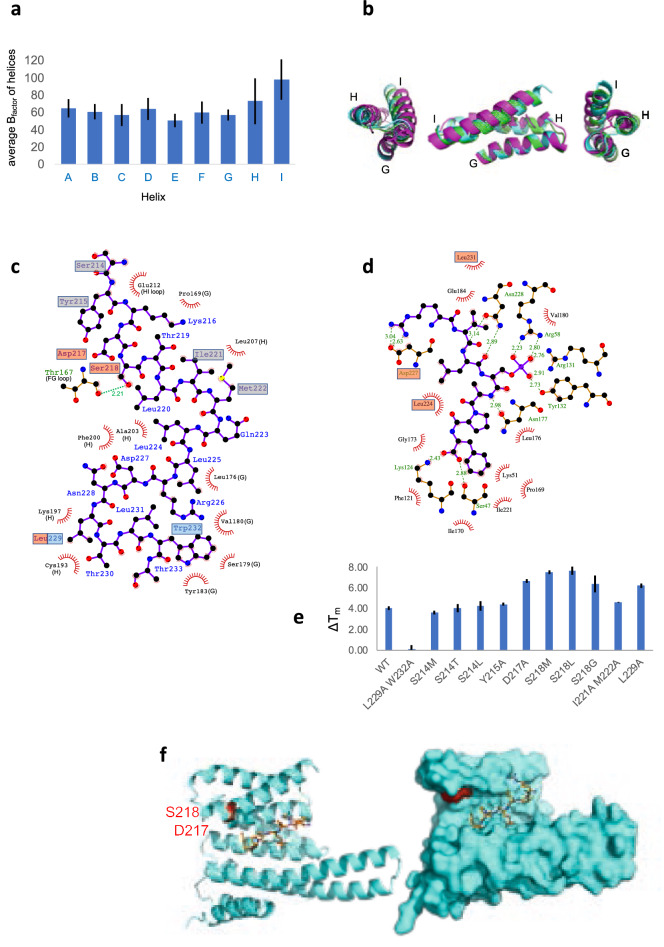
The flexibility of St14f helix I. (**a**) Average B-factors of each helix of St14f chain B. (**b**) Superposition of three chains of C-terminal helices G-I St14f in the crystal. They were fixed to helix G, which showed a relatively low B-factor. (**c**) Interaction diagram of St14f helix I and other helices generated by LigPlot+. The letters followed by the residue number show the helix or loop name. The hydrogen bonds are shown as the dotted line, while the hydrophobic contacts are represented by an arc with spokes. (**d**) Model of the StFDL1 peptide and St14f binding diagram based on the GF14c-OsFD1 structure. The residues involved in the interaction between the StFDL1 peptide and St14f helix I are represented in red. (**e**) Results of the thermal shift assay. The temperature changes caused by mutation are shown. (**f**) Mapping of the stabilized residues on the St14f surface that were revealed by thermal shift assay. The StFDL1 peptide is represented by an orange ball and stick model. Figures are prepared by PyMol 2.6 (http://www.pymol.org/pymol)^42^ and LigPlot+ 2.2 (https://www.ebi.ac.uk/thornton-srv/software/LigPlus/)^27^.

To evaluate the flexibility of St14f C-terminal helices, chain A, B, and C structures of the St14f free form were superposed using fixed helix G, which had the lowest B-factor in the C-terminal helices G-I (Fig. 4a). Helix I showed the largest conformational differences among the three chains. In comparison with other 14-3-3 structures, the 14-3-3 beta free, 14-3-3 beta complex, 14-3-3 sigma free, and GF14c complex were superposed very well with the chain B structure of the St14f free form (Supplementary Fig. S5). However, the 14-3-3 sigma complex was not superposed with others at helix I.

The B-factor of an atom is a potential measure to evaluate the flexibility because the B-factor is determined by its intrinsic flexibility and possible crystal packing effects^24^. In the St14f crystal, the solvent content of ~ 69% is larger than the typical value of 43%^25,26^, and thus, the contribution of the crystal packing seems smaller than the typical case. The average B-factors of each helix of the chain B structure (free form) of St14f were calculated and are depicted in Fig. 4b. The average B-factor of all helices A to G was 61 Å^2^, whereas the average B-factors of helices H and I were 73 and 98 Å^2^, respectively, suggesting that helices H and I are more flexible than other helices. The standard deviations of helices H and I were large, 26 Å^2^ (36%) and 24 Å^2^ (25%), respectively, suggesting that helices H and I contain both flexible and nonflexible residues. NMR experiments, such as ^1^H-^15^N hetero-nuclear Overhauser effect (NOE), were performed to evaluate the flexibility in solution, but they did not work well because of the lower sensitivity of NMR signals for the St14f homodimer with a larger molecular weight of ~ 60 kDa.

The structure of the free form of human 14-3-3 beta is drastically different from the complex form^20^. This structural difference suggests that the 14-3-3 protein is flexible. The MD simulation study of human 14-3-3 sigma suggests the presence of large-amplitude motion within the 14-3-3 molecule, i.e., helices A-D and G-I are rigid, but helices E and F are flexible^21^. In this study, we found that helix I is flexible, which has not yet been reported. There are some reasons why we could find this flexibility. First, our crystal structure is determined for the 14-3-3 free form. Most 14-3-3 structures are complex forms where the flexibility of helix I is not expected. Second, our crystal structure possesses the appropriate B-factor values to analyze the flexibility of helix I by chance. This flexibility is difficult to find because B-factors at medium resolution structures tend to be larger and less reliable than at higher resolution structures, and only 2 of 9 crystal structures of the 14-3-3 free form show clear differences (Supplementary Figs. S1 and S6, and Table S7). This situation is drastically improved when the "relative" B-factors are used for the analysis, and this flexibility is easily found in most 14-3-3 structures (Supplementary Figs. S1 and S7). Third, our crystal possesses the second-highest percentage of solvent content among the 14-3-3 free from crystals (Table 2). When the crystal of the 14-3-3 free form possesses a large solvent content, its B-factor of helix I is also large (Supplementary Figs. S1 and S6). This tendency could be explained by crystal packing (Supplementary Fig. S8). For example, for chain B of St14f free form, the decrease of the surface-accessible surface area of helix I due to crystal packing contacts was small ~ 11% (Supplementary Fig. S9 and Table S8). Furthermore, our St14f crystal structure was improved by Translation/Libration/Screw (TLS) refinement (Supplementary Fig. S6). The success of TLS refinement shows the presence of dynamic and static disorder due to the flexibility of crystalline proteins. For St14f, the B-factors of helices H and I are significantly reduced by TLS refinement. These data suggest that the flexibility of helix I is a general feature of 14-3-3 proteins.

**Table 2:** Matthews coefficients and solvent content of 14-3-3 free form crystal.

PDB	Organism		Matthews coefficient	solvent content (%)
7XBQ	*Solanum tuberosum* L	St14f	4.0	69.3
2BQ0	*Homo sapience*	Beta	2.7	54.6
1A4O	*Homo sapience*	Zeta	2.8	55.6
1YZ5	*Homo sapience*	Sigma	2.6	52.5
6TLG	*Homo sapience*	Sigma	4.5	72.6
5IQP	*Homo sapience*	Tau	2.3	46.9
2O8P	*Cryptosporidium parvum*		2.9	58.0
5BY9	*Giardia intestinalis*		3.7	67.1
5LVZ	*Lachancea thermotolerans*		2.7	54.4

### Helix I of St14f plays an important role in ligand recognition

The St14f free form and human 14-3-3 sigma complex showed the unique conformation of helix I, and then, the intra- and intermolecular interactions of helix I were analyzed by LigPlot^27^. For the St14f free form, the intramolecular interactions of helix I (S214-T233) were analyzed (Fig. 4c). Main chain interactions were excluded. Most intramolecular interactions of St14f helix I were hydrophobic with helix G or H. The interaction residues of helix I of St14f were P169, L176, S179, V180, Y183, C193, K197, F200, A203, L207, and E212. The intermolecular interactions of OsFD1 were analyzed for GF14c-OsFD1, which is an St14f-StFDL1 homolog. All detected interactions are depicted in Fig. 4d. The interaction residues of helix I of GF14c with OsFD1 were D227, L224, and L231, corresponding to D227, L224, and L231 on St14f.

The intermolecular interactions of St14f helix I with the StFDL1 peptide were examined by mutation. The mutation sites were selected from helix I and located away from the StFDL1 peptide binding site. The stability of the St14f-StFDL1 complex was evaluated by a thermal shift assay, and ΔTm (melting temperature difference) was calculated with and without the StFDL1 peptide (Table 3, Fig. 4e). The mutations at D217, S218, and L229 unexpectedly resulted in a larger ΔTm than WT, indicating the stabilization of the complex by these mutations (Figs. 4f and S1). The mutations at S214 and Y215 gave ΔTm values similar to WT, indicating similar stability. The L229 W232 double mutant exhibits no change with and without StFDL1, indicating no interaction with the StFDL1 peptide. The mutation of helix I located away from the StFDL1 peptide binding site improved the stability of the St14f-StFDL1 complex. This finding suggested that the flexibility of St14f helix I is involved in the stabilization of the complex between St14f and StFDL1.

**Table 3:** The difference in the melting temperature of St14f between free and complex with StFDL1 peptide measured by thermal shift assay.

St14f	ΔTm	SD
WT	4.03	0.20
L229A W232A	0.09^a^	0.38
S214M	3.60	0.16
S214T	4.01	0.39
S214L	4.26	0.47
Y215A	4.42	0.16
D217A	6.67^b^	0.17
S218M	7.48^b^	0.16
S218L	7.61^b^	0.39
S218G	6.37^b^	0.81
I221A M222A	4.63	0.03
L229A	6.22^b^	0.21

^a^No direct interaction with the StFDL1 peptide.

^b^Stabilized the complex with the StFDL1 peptide by mutation.

We also found the importance of helix I in the recognition of the binding partner. Masone et al*.* discussed that helix I may promote the correct conformation and location of the phosphorylated residue of the binding partner in the major binding groove of 14-3-3 zeta, consistent with our data showing the importance of helix I^28^. The presence of the flexibility of the binding partner has been discussed by Tugaeva et al*.*^29^, consistent with the discussion by Masone et al.^28^ It is noted that the helix I is located on the binding surface of B-Raf^30^, Nth1^31^, pHSPB6^32^, and Hd3a^17^, and plays an important role in these client protein bindings. Moreover, D228V mutation on helix I of human 14-3-3 gamma is related to the disease, developmental and epileptic encephalopathy 56^33^. The flexibility of helix I and/or the binding partners may allow the recognition of so many kinds of binding partners.

## Methods

### Expression and purification

The pCold GST vector contains a DNA fragment corresponding to St14f^34^. The construct was transformed into *E. coli* Rosetta (DE3). Cells were grown at 310 K in lysogeny broth medium containing 0.1 mg/mL ampicillin. Overexpression was induced by adding isopropyl β-D-thiogalactopyranoside (IPTG) to a final concentration of 0.3 mM at an optical density (OD) at 600 nm of 0.5–0.6. Cells were incubated at 288 K for 16 h under shaking at 100 rpm, harvested by centrifugation, and suspended in buffer [20 mM Tris–HCl pH 8.0, 200 mM NaCl, 1 mM dithiothreitol (DTT)]. The cell suspension was sonicated and centrifuged. The supernatant was loaded onto a Glutathione Sepharose 4B (GS4B) (GE Healthcare) column equilibrated with the same buffer (20 mM Tris–HCl pH 8.0, 200 mM NaCl, 1 mM DTT). GS4B resin was washed with the same buffer, and then the protein was eluted with a buffer (20 mM Tris–HCl pH 8.0, 200 mM NaCl, 1 mM DTT, 30 mM GlutathionepH 8.0). The elution was mixed with HRV 3C protease and dialyzed against 1 L of buffer (20 mM Tris–HCl pH 8.0, 150 mM NaCl, 1 mM DTT) at 277 K for 12 h for cleavage. The protein was rechromatographed using the GS4B affinity column to remove the residual affinity tag. The target protein was subjected to size exclusion chromatography equilibrated with buffer (20 mM Tris–HCl pH 8.0, 150 mM NaCl, 1 mM DTT).

### Crystal structure analysis

The purified St14f was dialyzed against 1 L of buffer (10 mM Tris–HCl pH 8.0, 1 mM DTT) and concentrated to 10 mg/mL (0.34 mM). The protein concentrations were determined using ultraviolet (UV) absorption measurements at 280 nm. The hanging drop vapor diffusion method is used for crystallization. Drops were made of 1 µL of protein and 1 µL of reservoir solution and suspended over 0.5 mL of reservoir solution at 277 K. St14f formed crystals of approximately 0.2 mm × 0.2 mm × 0.2 mm in dimension within 3 days using a reservoir solution of 12% (w/v) polyethylene glycol (PEG) 8000, 0.2 M ammonium tartrate pH 7. The crystal was sequentially transferred to 20% (v/v) ethylene glycol in the reservoir buffer using 2% increments and flash cooled in liquid nitrogen.

The diffraction dataset was collected at 100 K using an MX300HE CCD detector (Rayonix) on the BL44XU beamline at SPring-8 (Harima, Japan). The crystal-to-detector distance was set to 350 mm. Two hundred oscillations of 1° were collected with an exposure time of 2 s. The dataset was obtained at a wavelength of 0.9 Å. Reflections were integrated and scaled by HKL-2000^35^. Phasing of St14f was performed by molecular replacement with 14-3-3 protein from *Nicotiana tabacum* (PDB ID 1o9c; 94% sequence identity to St14f) using Phaser^36^. Structure refinement was performed with COOT^37^ and REFMAC5^38^ using the TLS refinement strategy^39,40^. TLS method extracts a linear combination of anisotropic translational (T), librational (L), and screw (S) motions from an analysis of the isotropic temperature factors of the atomic coordinates refined against X-ray diffraction data. The rigid-body motions of the peptide segments of potato 14-3-3 protein St14f were analyzed by the TLS method using the coordinates for St14f free form where the 233-residue amino acid sequence was partitioned into 20 TLS groups using the web-based server, http://skuld.bmsc.washington.edu/~tlsmd^38^. The structural model was evaluated using RAMPAGE^41^. The coordinate and structure factors have been deposited with the PDB with accession codes 7xbq (with TLS refinement). Data collection and refinement statistics including the space group are given in Table S1.

### HSQC spectrum

The ^1^H-^15^N-HSQC spectrum of the St14f free (red) and StFDL1 peptide complex (blue) at 298 K using a Bruker AVANCE III 950 MHz spectrometer equipped with a ^1^H/^13^C/^15^N TCI CryoProbe (Bruker BioSpin).

^15^N Lys-labeled St14f was prepared at 250 µM in a buffer containing 10 mM Tris, pH 7.5, 1 mM DTT, and 10% D_2_O. The complex sample was prepared with a final concentration of 1 mM StFDL1 peptide and 250 µM ^15^N Lys-labeled St14f. The spectrum was processed using the TopSpin software package (Bruker BioSpin). Assignments were carried out using single point mutations of St14f (Fig. S2).

### RDC measurement

RDCs were extracted from the chemical shift differences and resonance splitting measured from HSQC and TROSY spectra, which were collected on 0.5 mM St14f in 50 mM potassium phosphate buffer pH 6.8, 50 mM KCl, 10% D_2_O, with no phage and with 8 mg/mL Pf1 phage (ASLA BIOTECH). All experiments were conducted at 298 K and 950 MHz. Alignments of the samples containing Pf1 phage were confirmed by quadrupolar splittings of D_2_O signals in 1D ^2^H spectra, which were measured in conjunction with HSQC or TROSY measurements.

### Thermal shift assay

The St14f mutants were prepared by inverse PCR using KOD Plus (Toyobo). Purified St14f protein (10 mM HEPES pH 7.5 and 0.15 M NaCl buffer) was added to SYPRO Orange dye (stock concentration: 5000×) (Sigma). The final volume of each reaction was 20 µL, and the final concentration of St14f was 10 µM with no StFDL1 peptide and with 100 µM StFDL1 peptide. The 96-well plate was heated from 25 to 99 °C with a ramp rate of 1 continuous. The thermal shift assay was conducted in the Applied Biosystems real-time PCR instrument (API). The nonlinear fitting of thermal denaturation curves was performed to derive melting temperatures using Protein Thermal Shift Software.

## Supplementary Information

Supplementary Figure S1.

Supplementary Figure S2.

Supplementary Figure S3.

Supplementary Figure S4.

Supplementary Figure S5.

Supplementary Figure S6.

Supplementary Figure S7.

Supplementary Figure S8.

Supplementary Figure S9.

Supplementary Tables.

## Data Availability

The datasets and materials used and/or analyzed during the current study available from the corresponding author on reasonable request.
