# Supplementary figures and images for: Crystal structure of potato 14-3-3 protein St14f revealed the importance of helix I in StFDL1 recognition
Source: Sci Rep. 2022 Jul 8;12:11596. doi: 10.1038/s41598-022-15505-y (PMC9270373; doi:10.1038/s41598-022-15505-y)

Fig. 3c and 4f

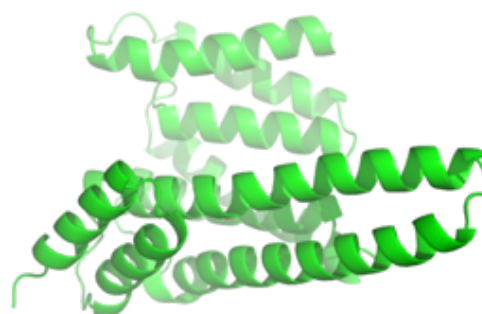

$y + 90^\circ$

Fig. 1b, 1c and 2

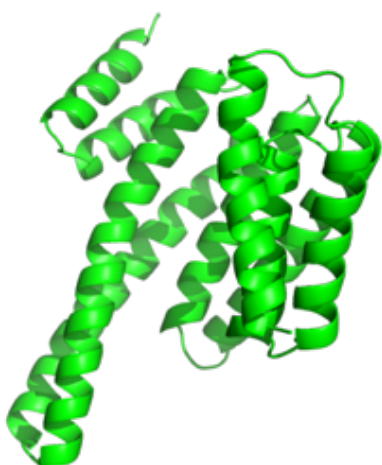

Fig. 1a and 1b

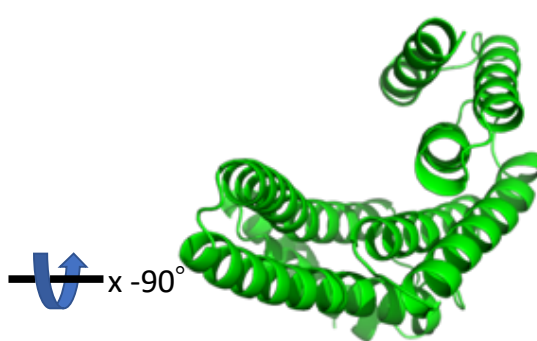

**Fig. S1.** The St14f structure view used in this paper.

Supplement: Supplementary file 1 — Supplementary Figure S1. [file 41598_2022_15505_MOESM1_ESM.pdf]

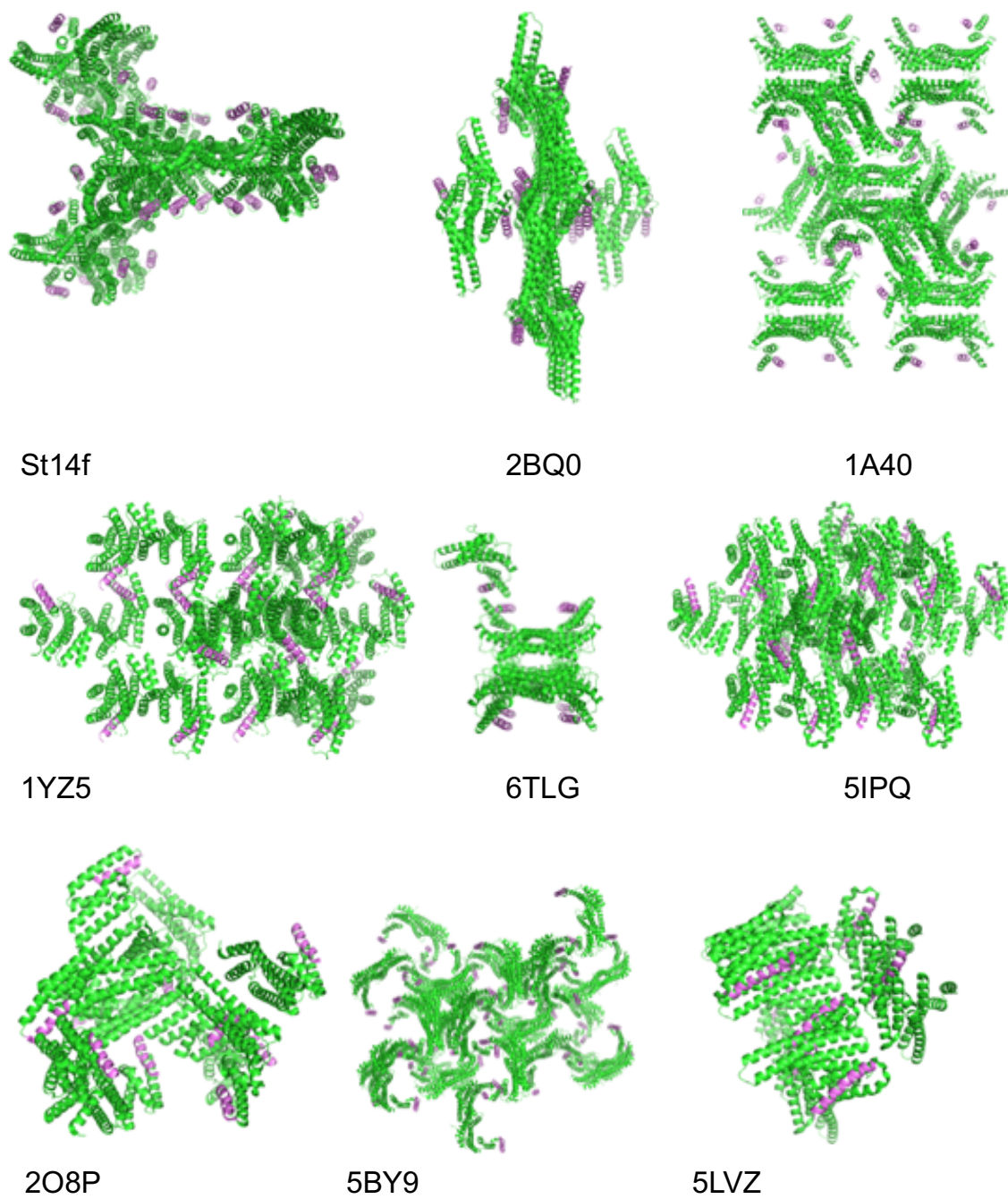

**Fig. S8.** Crystal packing of 14-3-3 proteins. Symmetry molecules are generated within 4 Å using PyMol.

Supplement: Supplementary file 8 — Supplementary Figure S8. [file 41598_2022_15505_MOESM8_ESM.pdf]
