# Supplementary material for: Crystal structure of potato 14-3-3 protein St14f revealed the importance of helix I in StFDL1 recognition
Source: Sci Rep. 2022 Jul 8;12:11596. doi: 10.1038/s41598-022-15505-y (PMC9270373; doi:10.1038/s41598-022-15505-y)
Supplement: Supplementary file 2 — Supplementary Figure S2. [file 41598_2022_15505_MOESM2_ESM.pdf]

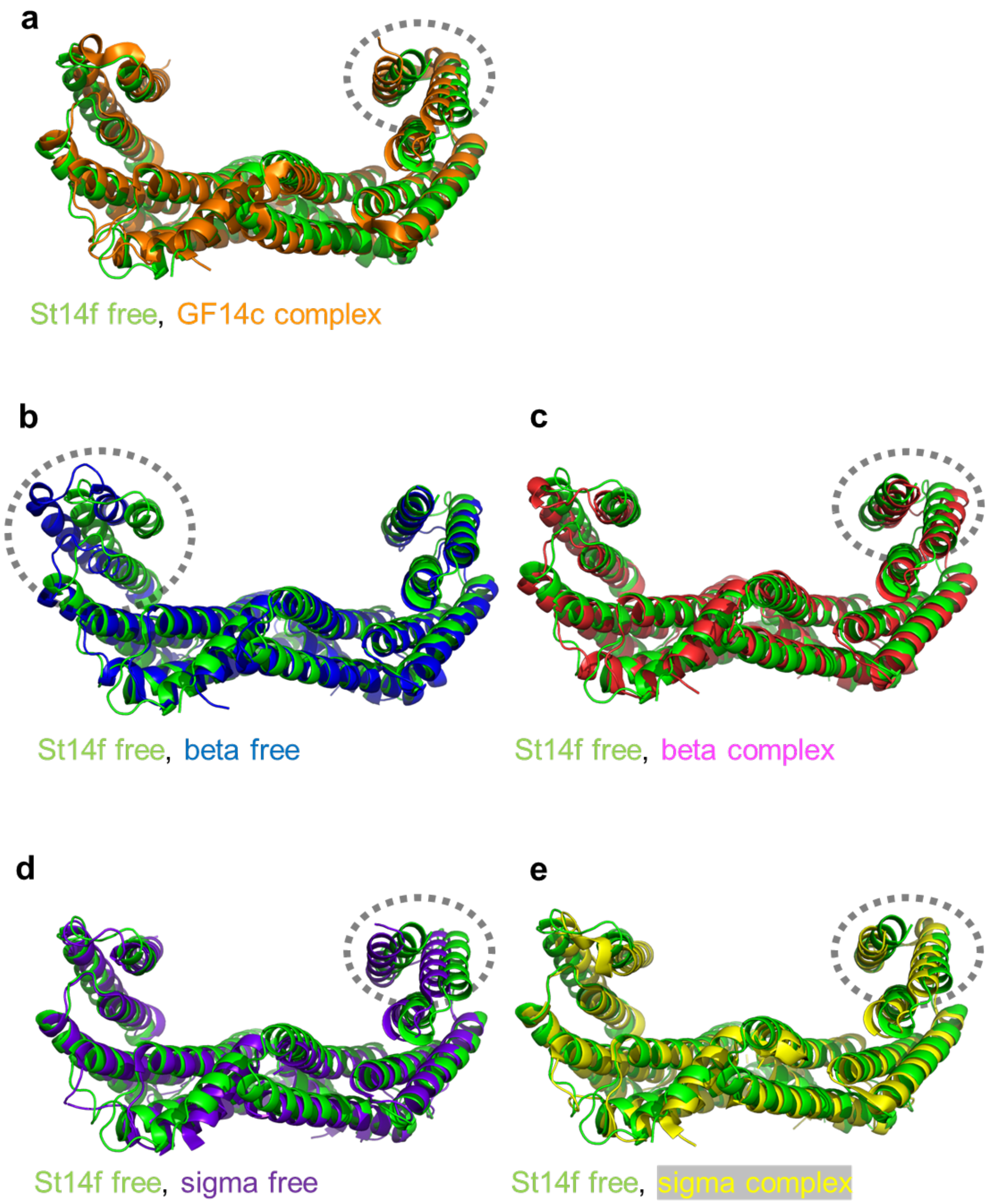

Fig. S2. Superposition of helices A-H between the St14f free form (chain AA, green) and other 14-3-3 protein dimers. (a) Rice GF14c complex form (orange) reported in PDB ID 3axy, (b) human 14-3-3 beta free form (blue) reported in PDB ID 2bq0, (c) human 14-3-3 beta complex form (magenta) reported in PDB ID 2c23, (d) human 14-3-3 sigma free form (purple) reported in PDB ID 1yz5, (e) human 14-3-3 sigma complex form (yellow) reported in PDB ID 1ywt. This figure is similar to Fig 2, indicating the significant displacements were found on both helices H and I.
