# Supplementary material for: Crystal structure of potato 14-3-3 protein St14f revealed the importance of helix I in StFDL1 recognition
Source: Sci Rep. 2022 Jul 8;12:11596. doi: 10.1038/s41598-022-15505-y (PMC9270373; doi:10.1038/s41598-022-15505-y)
Supplement: Supplementary file 3 — Supplementary Figure S3. [file 41598_2022_15505_MOESM3_ESM.pdf]

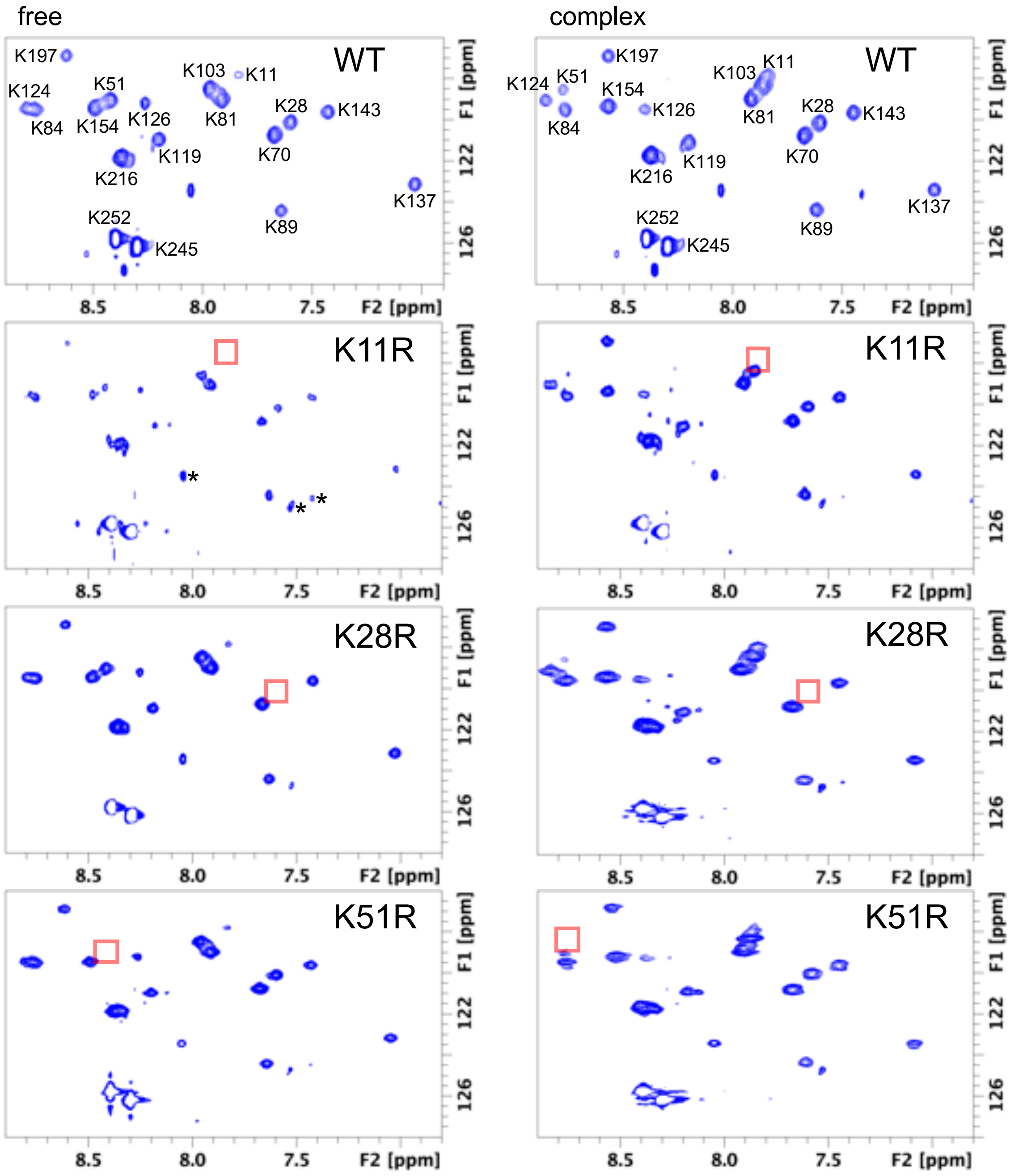
free
complex
WT
K197
K124
K51
K84
K154
K126
K103
K11
K81
K28
K143
K70
K119
K216
K137
K89
K252
K245
K11R
K28R
K51R
F1 [ppm]
F2 [ppm]
8.5
8.0
7.5
122
126

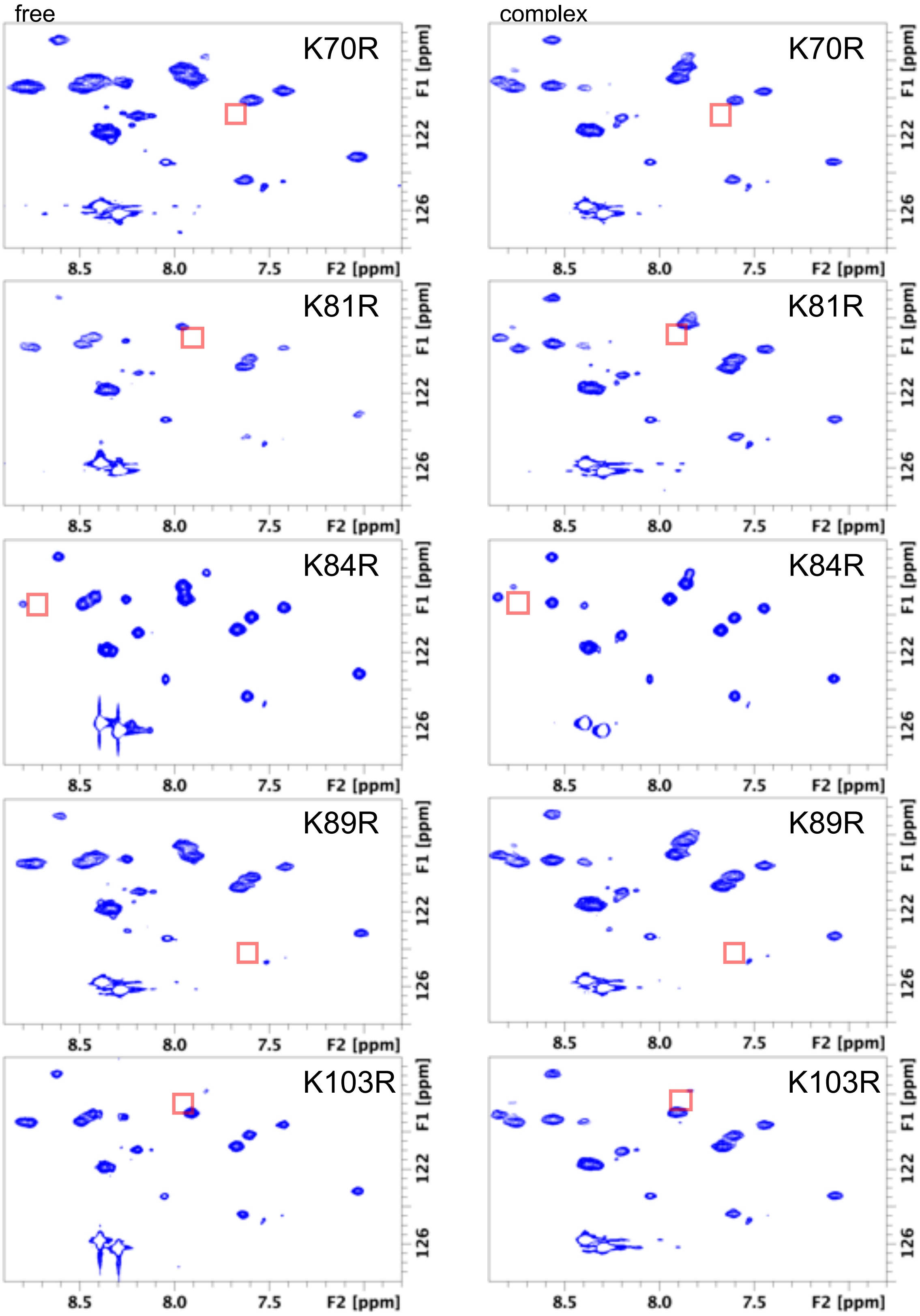

free
complex
K70R
K81R
K84R
K89R
K103R
F1 [ppm]
122
126
8.5
8.0
7.5
F2 [ppm]

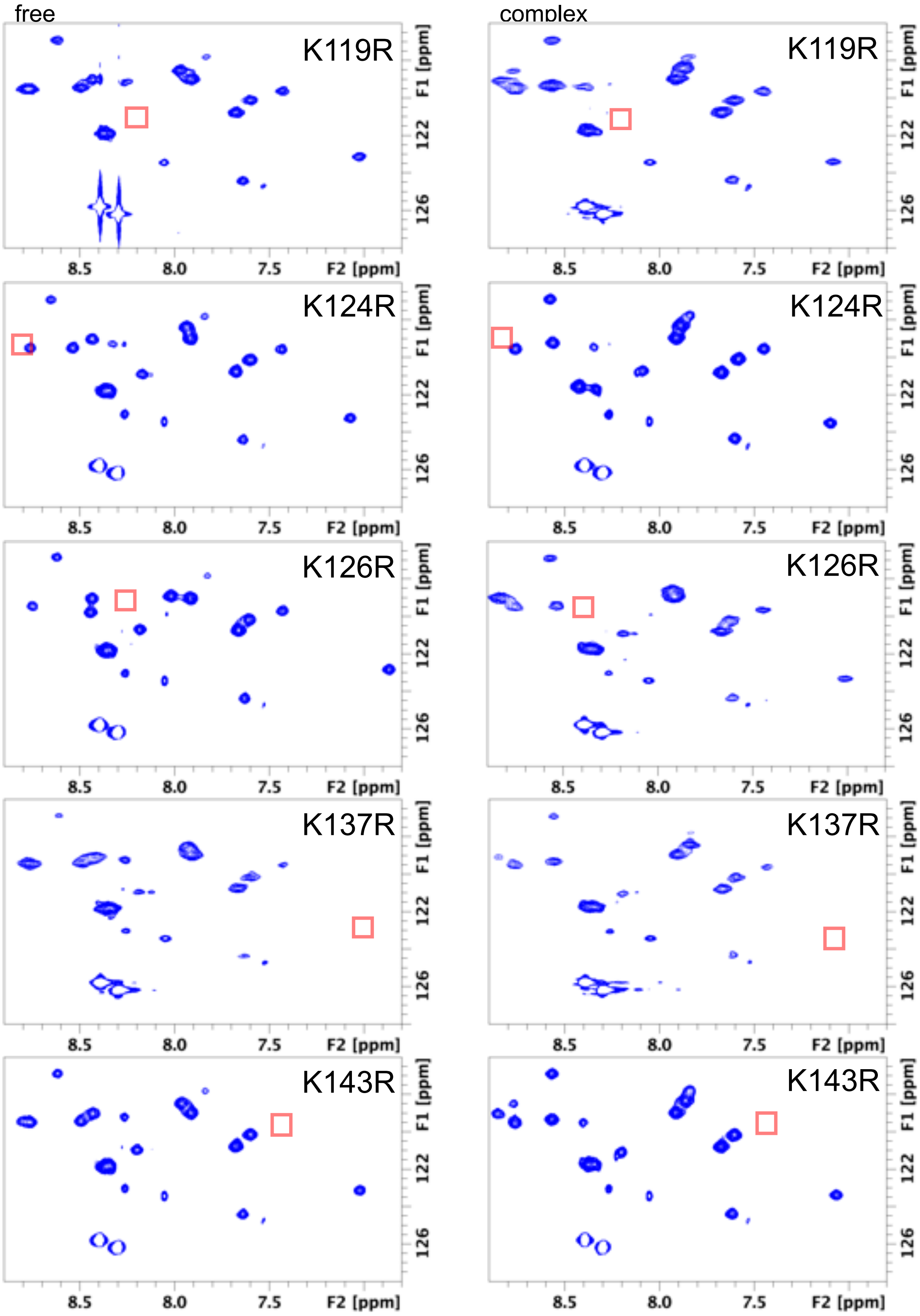
free
complex
K119R
K124R
K126R
K137R
K143R
F1 [ppm]
F2 [ppm]
122
126
8.5
8.0
7.5

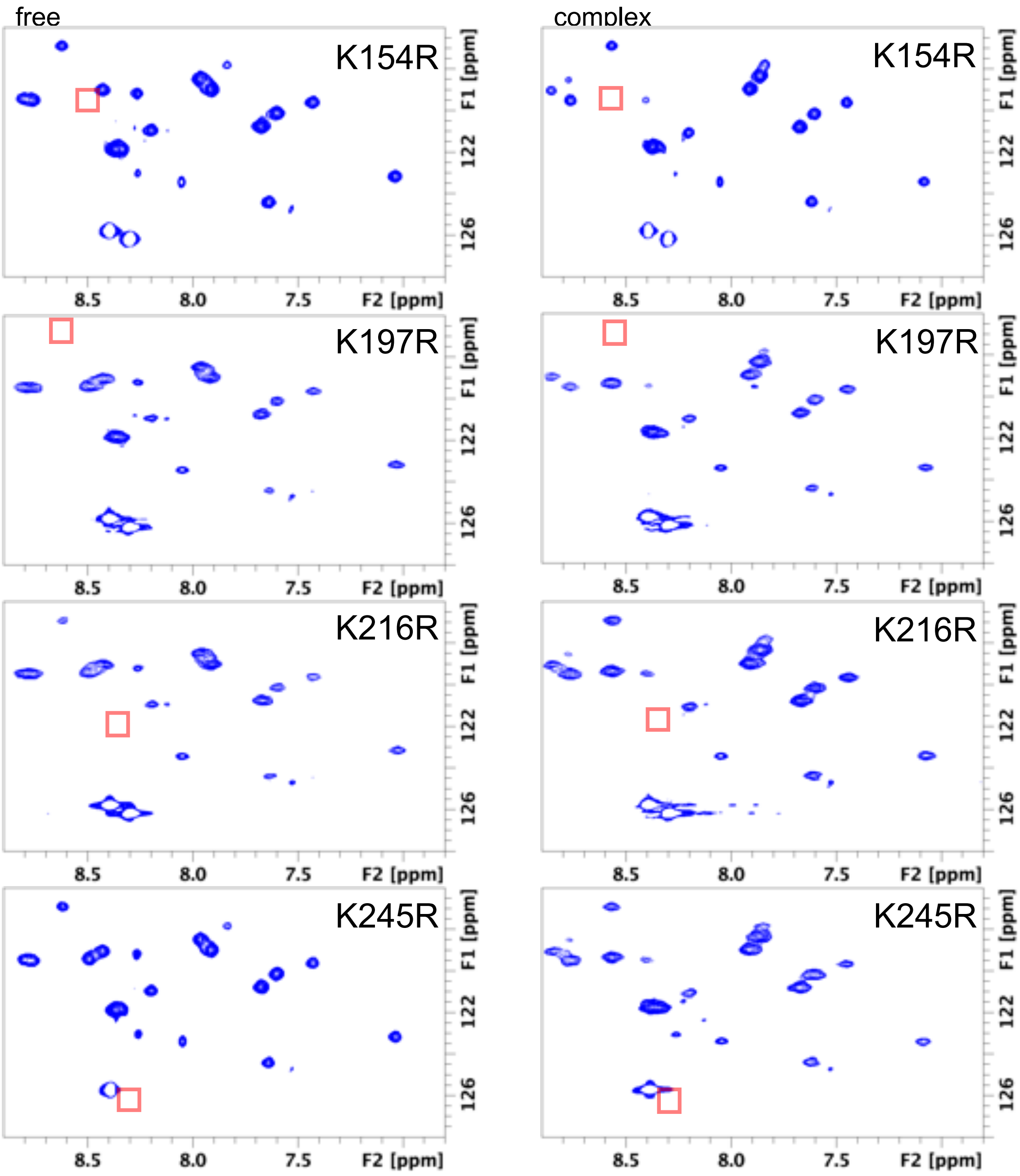

Fig. S3. Assignments of the ^{1}H-^{15}N-HSQC spectrum of ^{1}H-^{15}N Lys-labeled St14f free and StFDL1 peptide complex. HSQC spectrum of single-point mutants was measured and the lost peak was depicted by orange square.
