# Supplementary material for: Crystal structure of potato 14-3-3 protein St14f revealed the importance of helix I in StFDL1 recognition
Source: Sci Rep. 2022 Jul 8;12:11596. doi: 10.1038/s41598-022-15505-y (PMC9270373; doi:10.1038/s41598-022-15505-y)
Supplement: Supplementary file 4 — Supplementary Figure S4. [file 41598_2022_15505_MOESM4_ESM.pdf]

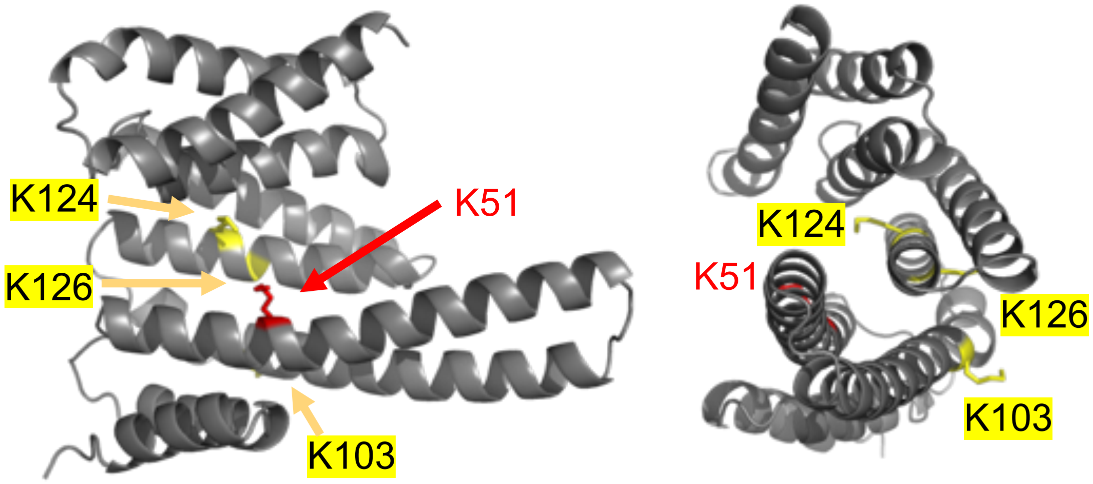

Fig. S4. Mapping of the Lys residues on the St14f, which exhibited the chemical shift changes of > 0.1. K51 showed the largest chemical shift on StFDL1 peptide binding, which was colored in red. Other Lys residues were colored in yellow. All of them were close to the positive charge groove.
