# Supplementary material for: Crystal structure of potato 14-3-3 protein St14f revealed the importance of helix I in StFDL1 recognition
Source: Sci Rep. 2022 Jul 8;12:11596. doi: 10.1038/s41598-022-15505-y (PMC9270373; doi:10.1038/s41598-022-15505-y)
Supplement: Supplementary file 5 — Supplementary Figure S5. [file 41598_2022_15505_MOESM5_ESM.pdf]

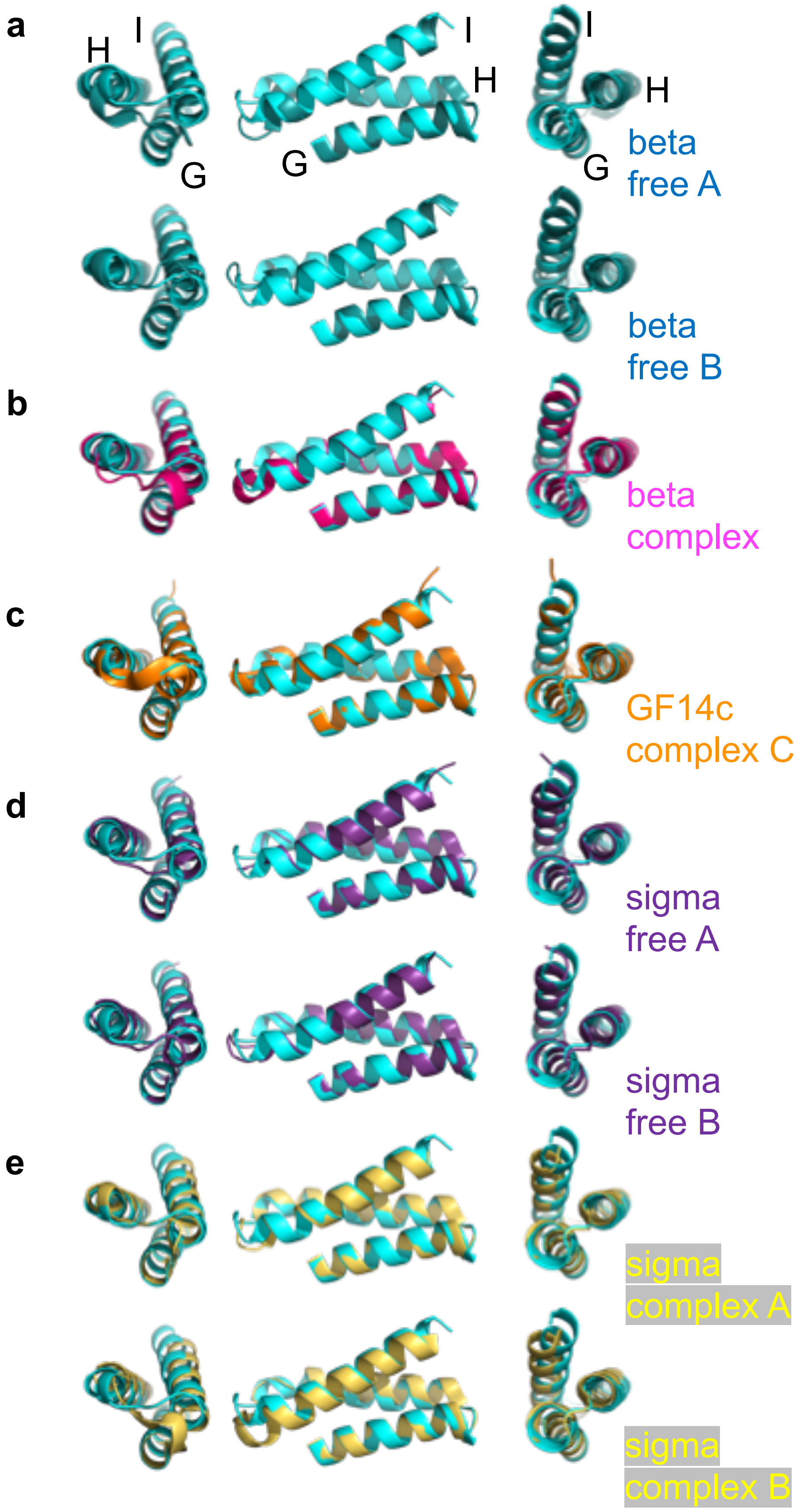
a
H
I
G
I
H
G
I
H
G
beta
free A
beta
free B
b
beta
complex
c
GF14c
complex C
d
sigma
free A
sigma
free B
e
sigma
complex A
sigma
complex B

Fig. S5. Superposition between C-terminal helices G-I of St14f free form (chain B, cyan) and other 14-3-3 protein dimers free or complex with peptide. The superposition was executed as the helix G was fixed. As the same with Fig. 2, (**a**) Human 14-3-3 beta free form (PDB ID 2bq0) was shown as a blue, (**b**) beta complex (PDB ID 2c23) as a magenta, (**c**) rice GF14c complex (PDB ID 3axy) as an orange, (**d**) human 14-3-3sigma free form (PDB ID 1yz5) as a purple, and (**e**) sigma complex (PDB ID 1ywt) as a yellow.
