# Supplementary material for: Crystal structure of potato 14-3-3 protein St14f revealed the importance of helix I in StFDL1 recognition
Source: Sci Rep. 2022 Jul 8;12:11596. doi: 10.1038/s41598-022-15505-y (PMC9270373; doi:10.1038/s41598-022-15505-y)
Supplement: Supplementary file 6 — Supplementary Figure S6. [file 41598_2022_15505_MOESM6_ESM.pdf]

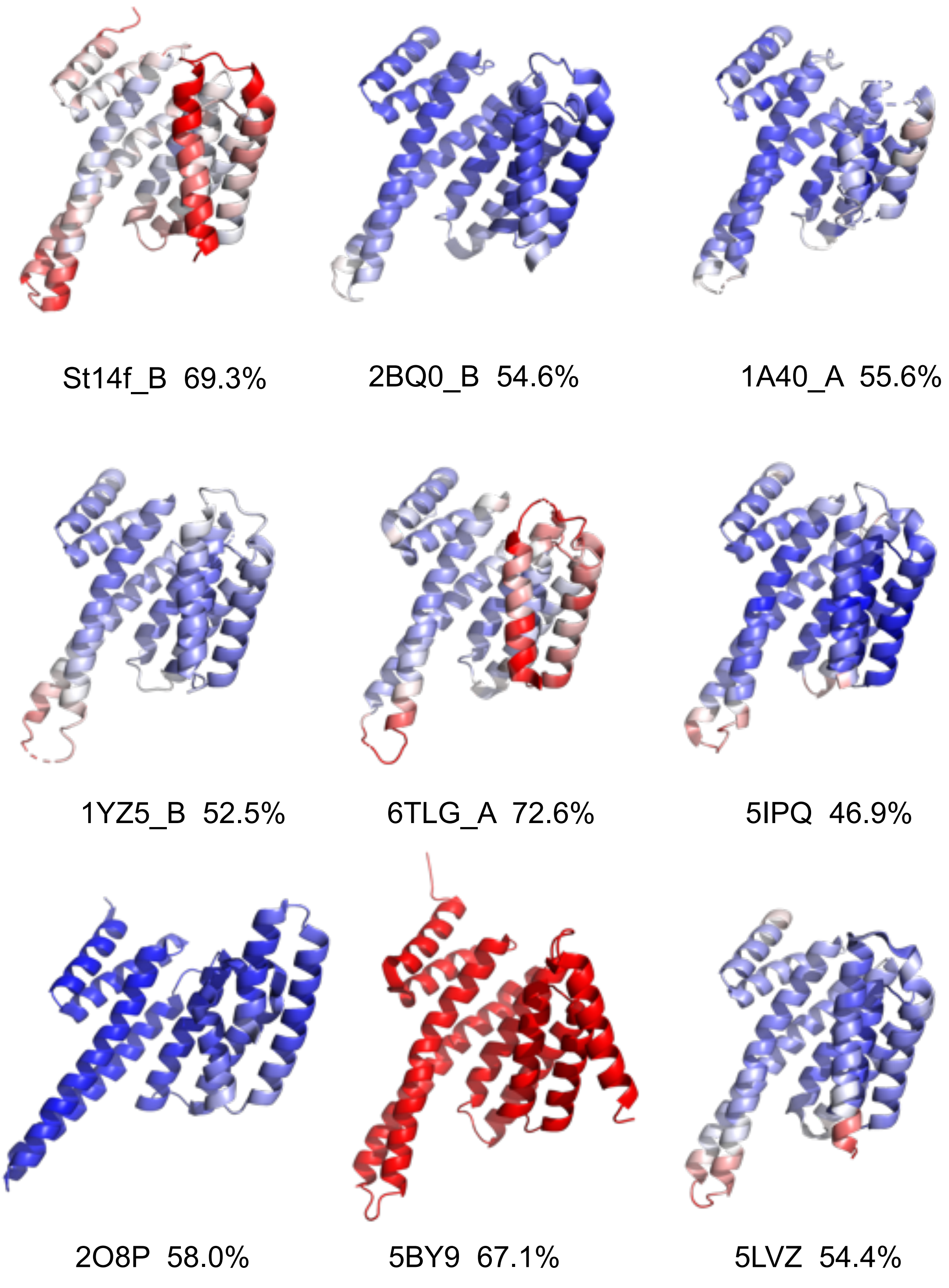

Fig. S6. Crystal structures of 14-3-3 free forms colored by B-factor blue-white-red (10-55-100 Å^2) using PyMol. PDB IDs and solvent contents were shown.
