# Supplementary material for: Crystal structure of potato 14-3-3 protein St14f revealed the importance of helix I in StFDL1 recognition
Source: Sci Rep. 2022 Jul 8;12:11596. doi: 10.1038/s41598-022-15505-y (PMC9270373; doi:10.1038/s41598-022-15505-y)
Supplement: Supplementary file 7 — Supplementary Figure S7. [file 41598_2022_15505_MOESM7_ESM.pdf]

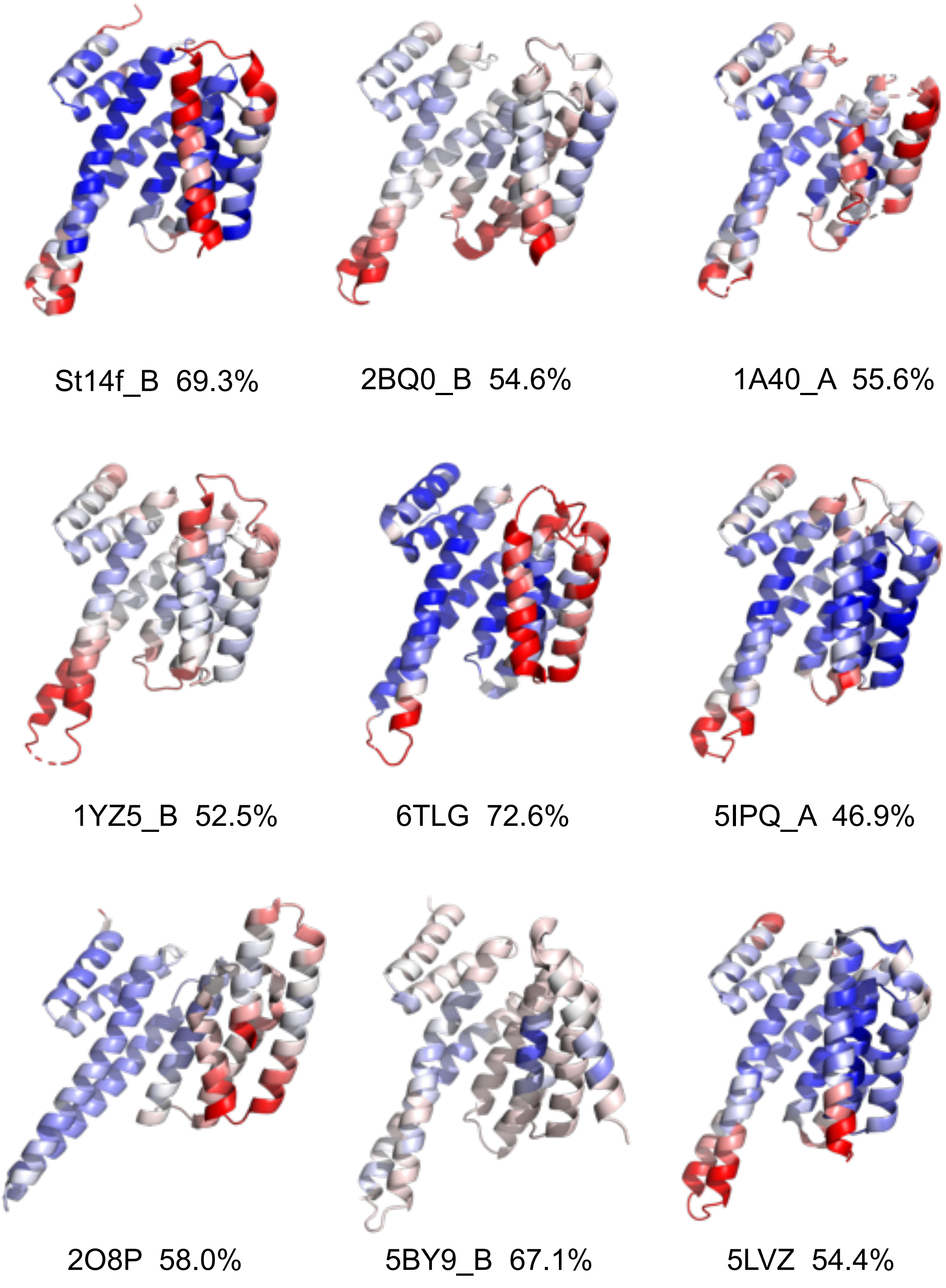

Fig. S7. Crystal structures of 14-3-3 free forms colored by relative B-factor blue-white-red (average – 20, average, average + 20 Å^2) using PyMol. PDB IDs and solvent contents were shown.
