# Supplementary material for: Crystal structure of potato 14-3-3 protein St14f revealed the importance of helix I in StFDL1 recognition
Source: Sci Rep. 2022 Jul 8;12:11596. doi: 10.1038/s41598-022-15505-y (PMC9270373; doi:10.1038/s41598-022-15505-y)
Supplement: Supplementary file 9 — Supplementary Figure S9. [file 41598_2022_15505_MOESM9_ESM.pdf]

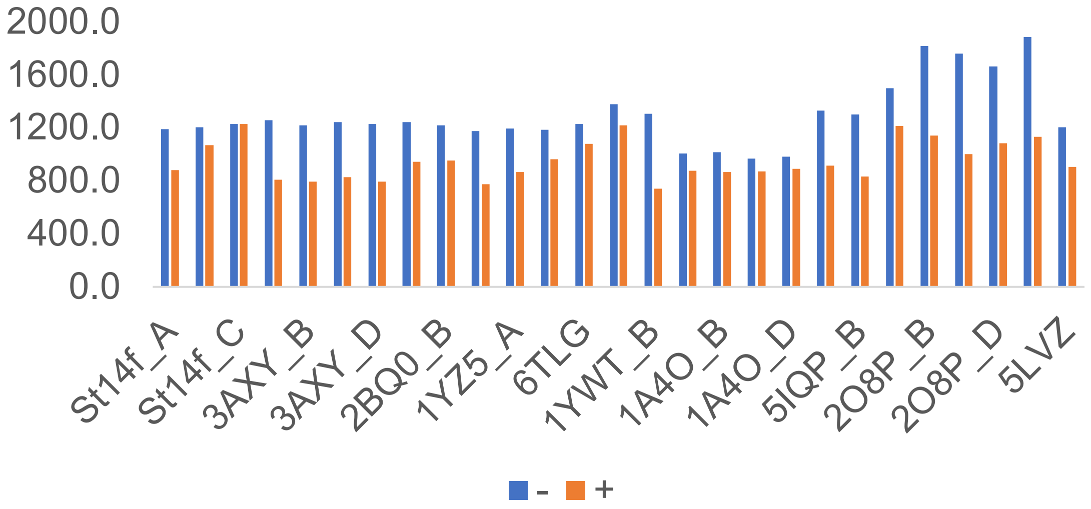

Fig. S9. Solvent-accessible surface area (SASA) values of helix I of 14-3-3 proteins. The SASA values shown by blue and red bars were calculated using PyMol with and without considering crystal packing, respectively.
