# Supplementary material for: Crystal structure of potato 14-3-3 protein St14f revealed the importance of helix I in StFDL1 recognition
Source: Sci Rep. 2022 Jul 8;12:11596. doi: 10.1038/s41598-022-15505-y (PMC9270373; doi:10.1038/s41598-022-15505-y)
Supplement: Supplementary file 10 — Supplementary Tables. [file 41598_2022_15505_MOESM10_ESM.pdf]

Table S1. Data collection and refinement statistics (Fig 1).

Wavelength (Å)	0.9
Temperature (K)	100
Detector	MX300HE
Crystal-to-detector distance (mm)	350
Rotation range per image (°)	1
Total rotation range (°)	200
Exposure time per image (s)	2
Space group	*C*2
a, b, c (Å)	230.2, 105.3, 61.0
α, β, γ (°)	90, 104.8, 90
Resolution range (Å)	47.6-2.45 (2.49-2.45)
Total No. of reflections	218717
No. of unique reflections	51160
Completeness (%)	99.7 (100.0)
Redundancy	4.3 (4.3)
<I>/<σ(I)>	28.8 (3.2)
R_{merge}	0.063 (0.442)
$CC_{1/2}$	0.981
Mosaicity	0.41-0.77
Reject	0.32
R_{factor}/R_{free}	0.25/0.30
R.m.s. deviations (length, Å / angle, °)	0.008/1.621
Ramachandran plot Favored/allowed/outlier (%)	95.5/4.2/0.3

Table S2. RMSD of St14f free form between chains A, B, and C (Fig 1b).

	Chain	RMSD (Å)
St14f	AB	0.520
	BC	1.192
	CA	1.075

Table S3. RMSD of C-terminal helices G-I between St14f free form and other 14-3-3 protein dimers (Fig 2).

Molecular 1	Molecular 2	RMSD (Å)
St14f free	GF14c complex	2.335
St14f free	beta free	5.438
St14f free	beta complex	1.261
St14f free	sigma free	3.207
St14f free	sigma complex	2.239

Table S4. ^1H and ^{15}N chemical shift values of lysine residues of St14f free and complex forms (Fig 3a and 3b).

Lys	free		complex		differences*
Res No	δ(^{1}H) (ppm)	δ(^{15}N) (ppm)	δ(^{1}H) (ppm)	δ(^{15}N) (ppm)	Δδ (ppm)
11	7.91	117.88	7.91	117.92	0.01
28	7.67	120.16	7.68	120.23	0.02
51	8.49	119.09	8.85	118.59	0.37
70	7.75	120.82	7.75	120.85	0.01
81	7.99	119.06	7.99	119.06	0.00
84	8.84	119.57	8.83	119.61	0.01
89	7.71	124.51	7.69	124.45	0.03
103	8.04	118.56	7.94	118.36	0.11
119	8.27	121.03	8.27	121.15	0.03
124	8.87	119.53	8.93	119.12	0.10
126	8.34	119.29	8.47	119.57	0.14
137	7.10	123.22	7.16	123.48	0.08
143	7.50	119.68	7.52	119.73	0.03
154	8.57	119.51	8.64	119.40	0.08
197	8.69	116.94	8.64	116.99	0.05
216	8.44	121.91	8.45	121.81	0.02
245	8.37	126.27	8.37	126.26	0.00
252	8.47	125.85	8.47	125.85	0.00

$*\Delta\delta = [\{\Delta\delta(^1H)\}^2 + \{\Delta\delta(^{15}N)/5\}^2]^{1/2}$

Table S5. Average B-factors of each helix of St14f free form chain B (Fig 4a).

Helix	Average B-factor (Å^2)
A	65 ± 11
B	61 ± 9
C	57 ± 13
D	64 ± 13
E	51 ± 8
F	60 ± 13
G	57 ± 7
H	73 ± 26
I	98 ± 24

Table S6. RMSD of helix I of St14f free form superposing helix G between chains A, B, and C (Fig 4b).

Chain	RMSD (Å)
AB	0.393
BC	2.259
CA	2.210

Table S7. Resolution, B-factor, and solvent content of 14-3-3 proteins (Fig S6).

Name	free / complex	PDB ID	Resolution (Å)	B-factor ($Å^2$)	R-free	R	Solvent Content (%)
Potato St14f	free	–	2.45	61.6	0.292	0.249	69.3
Potato St14f (TLS refinement)	free	7XBQ	2.45	45.0	0.281	0.240	69.3
Rice GF14c	complex	3AXY	2.40	32.1	0.276	0.227	65.3
Human 14-3-3 beta	free	2BQ0	2.50	28.2	0.273	0.214	54.6
Human 14-3-3 beta	complex	2C23	2.65	61.6	0.286	0.221	55.9
Human 14-3-3 sigma	free	1YZ5	2.80	37.7	0.292	0.219	52.5
Human 14-3-3 sigma	free	6TLG	2.40	39.6	0.254	0.212	72.6
Human 14-3-3 sigma	complex	1YWT	2.40	34.9	0.283	0.233	51.5
Human 14-3-3 zeta	free	1A4O	2.80	33.9	0.345	0.310	55.6
Human 14-3-3 tau	free	5IQP	2.60	NULL	0.239	0.186	46.9
Cryptosporidium parvum	free	2O8P	1.82	22.1	0.239	0.198	58.0
Giardia intestinalis	free	5BY9	4.00	117.6	0.233	0.233	67.1
Lachancea thermotolerans	free	5LVZ	1.95	36.5	0.223	0.189	54.4

Table S8. Solvent-accessible surface area (SASA) of helix I of 14-3-3 proteins (Fig S9).

PDB ID	Name	Chain	free / complex	SASA (Å^2) crystal packing –	SASA (Å^2) crystal packing +
7XBQ	Potato St14f (TLS refinement)	A	free	1191.3	879.7
		B	free	1206.4	1071.0
		C	free	1228.4	1228.4
3AXY	Rice GF14c	C	complex	1255.3	807.0
		D	complex	1219.0	791.8
		I	complex	1242.2	827.7
		J	complex	1228.4	792.8
2BQ0	Human 14-3-3 beta	A	free	1241.0	942.8
		B	free	1217.0	954.8
2C23	Human 14-3-3 beta	A	complex	1174.4	773.5
1YZ5	Human 14-3-3 sigma	A	free	1194.9	865.0
		B	free	1186.9	963.9
6TLG	Human 14-3-3 sigma	A	free	1227.4	1079.2
1YWT	Human 14-3-3 sigma	A	complex	1378.3	1219.8
		B	complex	1305.6	740.2
1A4O	Human 14-3-3 zeta	A	free	1005.0	876.8
		B	free	1014.0	865.8
		C	free	968.9	868.9
		D	free	981.9	888.9
5IQP	Human 14-3-3 tau	A	free	1327.6	914.7
		B	free	1300.5	831.4
2O8P	*Cryptosporidium parvum*	A	free	1497.9	1215.2
		B	free	1814.9	1143.7
		C	free	1757.4	1003.1
		D	free	1664.5	1085.6
5BY9	*Giardia intestinalis*	A	free	1882.7	1134.1
5LVZ	*Lachancea thermotolerans*	A	free	1202.5	903.6
